# “Controlled, cross-species dataset for exploring biases in genome annotation and modification profiles”

**DOI:** 10.1016/j.dib.2015.10.042

**Published:** 2015-11-10

**Authors:** Alison McAfee, Sarah Michaud, Leonard J. Foster

**Affiliations:** Department of Biochemistry and Molecular Biology, Centre for High-Throughput Biology and Centre for Sustainable Food Systems, University of British Columbia, 2125 East Mall, Vancouver, BC, Canada V6T 1Z4

**Keywords:** Apis mellifera, Proteomics, Mass spectrometry, Nanoelectrospray ionization, Proteome coverage

## Abstract

Since the sequencing of the honey bee genome, proteomics by mass spectrometry has become increasingly popular for biological analyses of this insect; but we have observed that the number of honey bee protein identifications is consistently low compared to other organisms [1]. In this dataset, we use nanoelectrospray ionization-coupled liquid chromatography–tandem mass spectrometry (nLC–MS/MS) to systematically investigate the root cause of low honey bee proteome coverage. To this end, we present here data from three key experiments: a controlled, cross-species analyses of samples from *Apis mellifera*, *Drosophila melanogaster*, *Caenorhabditis elegans*, *Saccharomyces cerevisiae*, *Mus musculus* and *Homo sapiens*; a proteomic analysis of an individual honey bee whose genome was also sequenced; and a cross-tissue honey bee proteome comparison. The cross-species dataset was interrogated to determine relative proteome coverages between species, and the other two datasets were used to search for polymorphic sequences and to compare protein cleavage profiles, respectively.

**Specifications** **Table**

**Table:** 

Subject area	Biology
More specific subject area	Shot-gun proteomics
Type of data	Mass spectrometry
How data was acquired	Easy-nLC1000 coupled to a Q-Exactive orbitrap
Data format	Raw data (RAW files), search results (TXT files)
Experimental factors	Comparison of proteome coverage between species; comparison of honey bee proteome coverage with and without accounting for sequence polymorphisms; comparison of protease activity across honey bee tissues
Experimental features	Protein samples were treated with dithiothreitol and iodoacetamide before trypsin digestion. Samples were desalted, then analyzed by nanoelectrospray ionization mass spectrometry (nESI-MS)
Data source location	Samples for the cross-species comparison were donated by researchers at the University of British Columbia, Vancouver, Canada. The bee for polymorphism analysis came from York University, Toronto, Canada. All other bee tissues originated from the apiaries at the University of British Columbia Farm
Data accessibility	ProteomeXchange (PXD002275)

**Value of the** **data**

• The mass spectrometry dataset represents the highest honey bee proteome coverage to date and provides peptide evidence to help refine the honey bee genome annotation.
• We describe a detailed example of creating a personalized proteome database for a honey bee, and the code provided here can be used to construct a personalized protein database for any organism with known SNPs.
• The controlled cross-species proteomes dataset is suitable for evolutionary and bioinformatic hypothesis testing.
• These datasets, while focused on honey bees, should allow others to test hypotheses around the relative completeness of genome annotation or differential modification profiles among the main model organisms.

## 1 Data

We provide here the data used to investigate why honey bee proteomics experiments tend to result in fewer protein identifications compared to other commonly studied species. We include the raw mass spectrometry data files for the cross-species comparison, the honey bee whose genome was also sequenced, and the cross-tissue comparison. We also include the MaxQuant protein search file of the tissue comparison data and the perl script used to generate the customized polymorphic protein database as Appendix A, Appendix A, respectively. We have provided a navigation table (Table 1) to aid readers in identifying the relevant data files to download from our ProteomeXchange submission.

## 2 Experimental design, materials and methods

### 2.1 Sample sources

For the species comparison experiment, *Mus. musculus* liver tissue was provided by Nicholas E. Scott, *Saccharomyces cerevisiae* was provided by Patrick Chan, *Drosophila melanogaster* was provided by Carol Pollock, *Caenorhabditis elegans* was provided by George Chung. The *Apis mellifera* sample for polymorphism analysis originated from York University in the summer of 2010, courtesy of Amro Zayed and Brock A. Harpur. All other *A. mellifera* samples were collected the University of British Columbia apiary in the summer of 2014.

## 3 Cross-species comparison of proteome coverage

### 3.1 Sample preparation

**Fig. 1:**
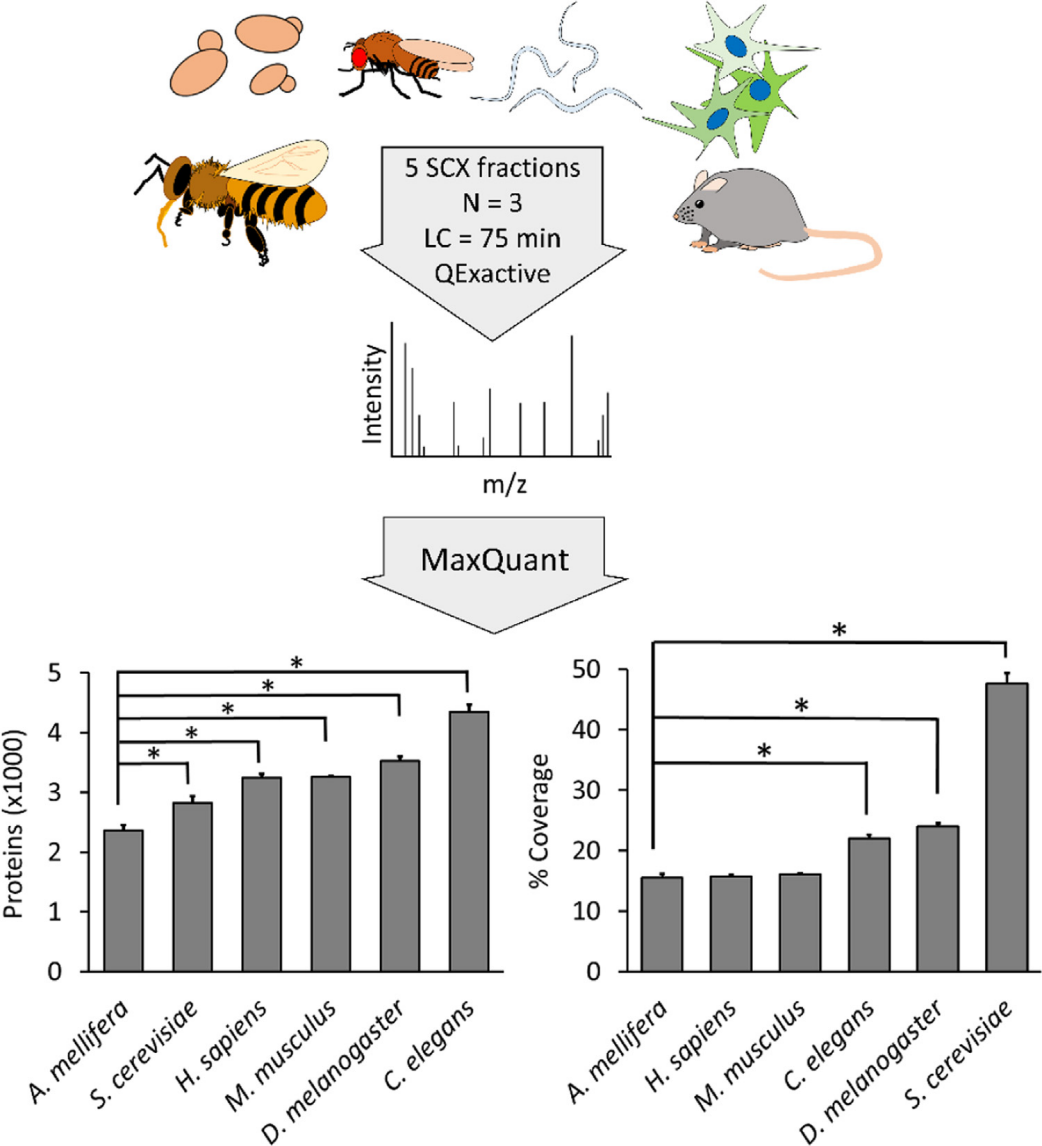
Confirming low proteome coverage in honey bees. Proteome coverage across several well-studied species (*Apis mellifera*, *Saccharomyces cerevisiae*, *Homo sapiens*, *Drosophila melanogaster*, *Caenorhabditis elegans* and *Mus musculus*) was compared in a controlled experiment. Sample preparation techniques were identical wherever possible and all samples were run sequentially on the same Q-Exactive mass spectrometer and liquid chromatography system. Left graph: significantly fewer honey bee proteins were identified compared to all other species. Right graph: honey bees showed the lowest canonical proteome coverage overall. * *P*<0.05; Student׳s *T*-test, df=4.

To provide experimental evidence for low protein identification rates in bees, we compared proteome coverage across six model systems in a controlled experiment (Fig. 1; Table 1; data files starting with “species_comparison”). All samples were processed and analyzed in biological triplicate. Protein was extracted in 50 mM ammonium bicarbonate (plus 1% sodium deoxycholate), with the aid of ceramic beads for mechanical disruption of coarse tissues. Clarified lysates were heated to 99 °C for 5 min, quantified using a bicinchoninic assay (Pierce), then were reduced (1 µg dithiothreitol per 50 µg protein), alkylated (5 µg iodoacetamide per 50 µg protein) and digested (1 µg porcine modified trypsin per 50 µg protein) overnight at 37 °C. Samples were acidified with 1% trifluoroacetic acid until pH<2.0 and the precipitated deoxycholatic acid was pelleted by centrifuging at 16,000*g*. Peptides in the supernatant were desalted using a high-capacity C18 STAGE-tip [2] and split into five fractions by strong cation exchange as described previously [3].

### 3.2 Data acquisition

The cross-species analysis was done in biological triplicate and equal amounts of protein were analyzed from each, all with equal amounts of instrument time. Fractionated peptides were analyzed by nLC–MS/MS using a nanoflow HPLC (Easy-nLC1000) coupled to a Q-Exactive mass spectrometer (Thermo). For each fraction, peptides were injected into the LC and loaded with Buffer A (0.5% acetic acid) onto an in-house packed fused-silica (5 μm Aqua C18 particles; Phenomenex) fritted trap column (2 cm long, 100 μm I.D., 360 μm O.D., 5 μL/min flow rate), then resolved on a reverse phase 75 μm I.D. fused silica, in-house packed 30 cm analytical column (ReproSil C18, 3 μm particle size; Dr. Maisch) using a 75 min linear gradient (90 min total run time) at a flow rate of 250 μl/min from 5% to 35% Buffer B (acetonitrile, 0.5% acetic acid), followed by a 15 min wash at 95% Buffer B. Instrument acquisition parameters included a 1% underfill ratio, intensity dependent MS/MS at 1.7e5 intensity threshold, and the instrument was set to scan from 300 to 2000 m/z with a 30 s dynamic exclusion time.

### 3.3 Search parameters

For each species, raw files were analyzed in a single MaxQuant search (version 1.5.2.8) [4] with all parameters left as default, except “match between runs” was enabled and data files were assigned their respective groups and fractions. The most recent honey bee Official Gene Set (OGS) protein database was downloaded from www.beebase.org (OGSv3.2). All other databases were downloaded from Uniprot proteomes (www.uniprot.org) on Feb. 5th, 2014.

## 4 Cross-tissue comparison of protease activity

We extracted protein from tissues with varying degrees of endogenous proteases (antennae, legs and digestive tract). The protein was processed and analyzed as described above, except it was not fractionated and the deoxycholatic acid was removed by centrifuging through a 0.6 µm filter (Sartorius Stedim Biotech). 5 µg of peptides was injected (based on protein-level quantitation) per sample. The data was searched as above, but with both trypsin specificity and no enzyme specificity. In the absence of substantial protease activity, searches with no enzyme specificity typically decrease the total number of matched peptides owing to the increase in search space, except when endogenous protease activity is abundant. This dataset represents the highest coverage of the honey bee proteome (protein identifications are provided as Supplementary file 1).

## 5 Impact of accounting for genetic diversity

Proteins from a single adult worker bee were extracted from the head essentially as outlined above but in 6 M urea lysis buffer [5] rather than ammonium bicarbonate and deoxycholate. Samples were quantified using a Bradford assay, then digested and analyzed on a Q-Exactive as described above, but with a three hour total analytical gradient. The data was searched using Mascot v2.5 with TrypsinMSIPI specificity and the decoy search option was used to filter for 1% false discovery rate. The protein database used in this search included this bee׳s specific SNPs, which was produced following the methods outlined in [6]. We have included our perl script used to generate the protein database as Supplementary file 2.

## Figures and Tables

**Table 1:** Description of the dataset.

Database(s)	Result file(s)	Data file(s)	Experiment
uniprot-(.*).fasta; amel_OGSv3.2_pep.fa	[bee|HeLa|fly|mouse|worm| yeast]_peptides.txt [bee|HeLa|dro|mouse|worm |yeast]_summary.txt	species_ (.*).raw	“Cross-species comparison of proteome coverage”
amel_OGSv3.2_pep.fa	Supplementary material	tissue_ (.*).raw;	“Cross-tissue comparison of protease activity”
finalApisSNPPersonalizedDB.fasta	customOGS_peps.txt	customOGS_bee_head_3hr.raw	“Impact of accounting for genetic diversity”
